# Inorganic carbon enrichment does not increase production of polyunsaturated aldehydes in a pelagic and benthic diatom

**DOI:** 10.1371/journal.pone.0328171

**Published:** 2025-07-10

**Authors:** Jeremy P. Johnson, Karin L. Lemkau, Ian W. Parker, Michael Brady Olson

**Affiliations:** 1 Departments of Biology and Chemistry, Western Washington University, Bellingham, Washington, United States of America; 2 Department of Chemistry, Western Washington University, Bellingham, Washington, United States of America; 3 Department of Biology, Western Washington University, Bellingham, Washington, United States of America; Universite Libre de Bruxelles, BELGIUM

## Abstract

Seasonal upwelling in coastal environments supports high primary production by increasing concentrations of inorganic nutrients in the euphotic zone. Diatoms typically dominate planktonic primary production and community composition during seasonal upwelling, especially in temperate ecosystems. Some diatoms elevate their competitive fitness by producing polyunsaturated aldehydes (PUAs). These phytochemicals act to reduce the fecundity of their grazers by reducing sperm motility, lowering egg production and viability, and delaying embryo development, reducing diatom consumptive pressure. While research into the mechanisms driving PUA production includes bottom-up factors (i.e., nutrient availability), few studies have explored how dissolved carbon dioxide (*p*CO_2_) concentration affects PUA production. In this study, we analyzed the production of bioactive PUAs (2,4-heptadienal, 2,4-octadienal, and 2,4-decadienal) in two diatom species found in the Salish Sea, an inland sea of the North Pacific ecosystem, under varying *p*CO_2_ concentrations that are experienced during seasonal upwelling events. We found that elevated *p*CO_2_ concentration caused an increase in carbon uptake in the diatoms, but did not lead to more PUA production, and at times caused a decrease in production. Our results suggest that carbon enrichment does not elevate the chemically defensive capabilities of diatoms by way of elevated PUA production.

## Introduction

Seasonal upwelling in temperate coastal environments supports high growth rates [1] and blooms of diatoms [2] through delivery of high concentrations of inorganic nutrients to surface waters [3–5]. Diatoms contribute up to 70% of net primary production in coastal upwelling systems [6]. This primary production supports high secondary production of pelagic grazers like copepods [7], and through strong benthic-pelagic coupling, benthic consumers such as crustaceans and mollusks [8]. Diatoms show high competitive fitness through bottom-up and top-down processes, including, but not limited to, high nutrient uptake [9] and anti-herbivory mechanisms [10–15].

Some diatoms can produce cytotoxic and allelopathic biomolecules that increase their competitive fitness [16,17]. One suite of molecules with known biotoxic effects are polyunsaturated aldehydes (PUAs). PUAs are produced upon cell damage (e.g., grazing) whereby polyunsaturated fatty acids (PUFAs) like eicosapentaenoic and hexadecatrienoic acid are oxidized using lipoxygenase enzymes [18]. PUAs can reduce predator reproductive output through maternal effects [19]. For example, PUAs, ingested through diatom consumption, can lower fecundity in female copepods by reducing egg production and viability, as well as embryo survival and larval development [20]. PUAs can also impact non-copepod diatom grazers, and have been shown to reduce benthic invertebrate sperm motility [21] and negatively impact sea urchin embryo and larval development [22,23] and oyster hemocyte cytoskeleton organization [24]. PUAs can also affect vertebrate cell functioning, including inducing apoptosis in human cancer cell lines [25].

PUAs are produced by many pelagic and benthic diatoms [26–28]. Within PUA producers, production can vary with nutrient concentrations [29] and growth phase of the population [30]. Growth phase studies have shown that PUA concentrations increase with bloom age [31]. Nutrient limitation studies on PUA production have produced conflicting results, with PUA dynamics linked to specific nutrients; silica and phosphorus limitation caused an increase in PUA production [29], whereas production decreased in nitrogen-limited diatoms [32]. Nitrogen limitation directly affects the availability of nitrogen-containing lipoxygenase enzymes present within diatom cells, thus reducing the cellular machinery needed to generate PUAs from precursor molecules [18].

Diatoms may also alter their capacity to produce PUAs through the production of PUFA precursor molecules. Given the low binding efficiency of RubisCO at modern CO_2_ concentrations in today’s oceans [33], diatoms use energy-intensive carbon-concentrating mechanisms (CCMs) to increase intercellular CO_2_ concentrations around RubisCO [34,35]. However, the need for CCMs declines in active upwelling regions where dissolved carbon dioxide (*p*CO_2_) concentrations are elevated [36]. In areas like the North Pacific Salish Sea, seasonal upwelling increases ambient *p*CO_2_ concentrations from ~400 μatm to upwards of 1200 μatm during upwelling events [37,38]. By increasing the availability of *p*CO_2_ for photosynthesis and reducing the need for CCMs, diatoms may utilize net photosynthesis to produce carbon-based secondary metabolites, including PUFA molecules and their resulting PUAs.

The connection between *p*CO_2_ and PUA production has been investigated previously under constant high *p*CO_2_ conditions, indicative of global ocean acidification rather than from sporadic *p*CO_2_ additions during upwelling events. While one study found that high but static *p*CO_2_ concentration did not change precursor PUFA concentration [39], a similar study found a decrease in PUA production with increased *p*CO_2_ concentration [40]. In addition, these studies only analyzed PUA production response in a pelagic diatom. No study has assessed the impact of episodic elevated *p*CO_2_ concentrations experienced during upwelling events or examined impacts on PUA production by benthic diatoms.

In the current study, we compare the production of PUAs under varying *p*CO_2_ concentrations observed during upwelling events along the west coast of the United States. We used two representative pelagic and benthic diatom species found in the Salish Sea to determine whether production of three commonly produced, bioactive PUAs change in concentration under simulated upwelling. We monitored production of 2,4-heptadienal, 2,4-octadienal, and 2,4-decadienal, hereafter referred to as heptadienal, octadienal, and decadienal. This study will improve our understanding of the factors that select for PUA production and, in general, diatom chemical ecology under varying environmental conditions.

## Methods

### Diatom cultivation

The benthic diatom *Fragilariopsis pseudonana* [41] was isolated from a mixed diatom assemblage collected from the Salish Sea near Bellingham, Washington (48.7193°N, 122.5158°W), while the pelagic diatom *Skeletonema marinoi* was purchased from the National Center for Marine Algae and Microbiota. Following Washington State Department of Fish and Wildlife guidelines, no permits were required for the collection of seawater and algal samples. Diatom cultures were maintained in polycarbonate bottles (125 mL) using autoclaved filtered seawater (AFSW) amended with F/4 growth medium. Cultures were grown in an environmental incubator under a 11:13 light:dark cycle at 15°C. Once per week, cultures were homogenized by gentle mixing, volumes were reduced by 75%, and cultures refilled with growth medium-amended AFSW.

### *p*CO_2_ experiment

To cover the range of *p*CO_2_ concentrations that would be experienced during pre- and post-upwelling events in the Salish Sea, three concentrations were used: 400 (ambient; non-upwelling), 800 (medium), and 1200 μatm *p*CO_2_ (high) [37,38]. Inorganic carbon was delivered into the experimental system according to [42]. Briefly, ambient air was scrubbed of CO_2_ and subsequently mixed with research grade CO_2_ gas (99.9% CO_2_, AirGas) in known concentrations using mass flow controllers. The gas mixture was bubbled into carboys containing AFSW amended with F/4 growth medium for at least 24 hours, with this liquid hereafter referred to as the equilibrated media.

For each *p*CO_2_ treatment, 500 mL of equilibrated media was inoculated with 5 mL of dense diatom cultures (174.54 µg chlorophyll-*a*/L for *S*. *marinoi* and 398.61 µg chlorophyll-*a*/L for *F*. *pseudonana*) in quadruplicate replication, sealed with Teflon tape to prohibit gas exchange, and placed randomly in the environmental incubator. Within each treatment, triplicate blank bottles containing no diatoms were included to ensure changes in pH and dissolved inorganic carbon (DIC) over the duration of the experiment could be attributed to diatom metabolism and were not the result of outgassing. Each day, bottles were inverted to ensure homogenous *p*CO_2_ concentrations and were subsequently randomly placed back into the incubator under the environmental conditions described above. This sealed bottle (i.e., no air-sea gas exchange) design was chosen to replicate a water parcel enriched in dissolved inorganic carbon and nutrients, but not in contact with the atmosphere, as observed during or immediately after an upwelling event. The experiment lasted 6 days which, based on preliminary experiments, was sufficient to ensure the cultures reached stationary growth.

### Carbonate chemistry

One bottle from each treatment was randomly selected for monitoring of pH and DIC throughout the entire experiment, while all other treatment bottles remained sealed for the duration of the experiment. To monitor carbon uptake, on Days 0, 3, and 6, pH and DIC were assessed using a UV-VIS flame spectrophotometer (Ocean Optics S) and a DIC Analyzer (Apollo SciTech AS-C3) according to [43]. For pH analysis, water samples were warmed to 24.95°C and transferred to a 5 cm jacketed cuvette using a syringe to minimize gas exchange. The samples received two injections of 20 µL *m*-cresol dye and absorbance was measured at 434, 578, and 730 nm wavelengths after each injection. pH was also monitored daily using a ThermoScientific Orion Star A121 pH probe, though no trends were observed that are not already reflected in our UV-vis pH measurements. For DIC analysis, 20°C samples were injected into the DIC analyzer with 10% phosphoric acid and measured for total DIC using a calibration curve created from reference solution (CRM, Batch 179, Dickson, Scripps Institute of Oceanography). Temperature and salinity at time of sampling were measured using the Orion pH probe. All spectrophotometric and DIC data was input into CO2SYS [44] to calculate pH (total scale), total DIC, and *p*CO_2_ (μatm).

### Chlorophyll-*a* Standardization

Chlorophyll-*a* was used to standardize PUA production across treatments. Uniformly mixed culture aliquots (10 mL) were filtered onto GF/C filters, and filters placed into test tubes containing 90% acetone. Test tubes containing filters were stored at −20°C in darkness for 24 hours to allow for chlorophyll extraction. Chlorophyll-*a* was quantified using a Trilogy fluorometer on acidification mode by measuring fluorescence before and after acidification with 1 M hydrochloric acid [45]. Growth rates (d^-1^) of diatom cultures were calculated using pre- and post-experiment chlorophyll measurements assuming exponential growth.

### PUA Analysis

We define PUA production as the mass of PUAs capable of being produced by the diatoms before cell disruption, or the mass of PUAs that would be released into the water column if all diatom cells were naturally disrupted through predation. PUA molecules were extracted and quantified following previously described methods [28]. Briefly, diatom cultures were filtered, disrupted by freezing, and the released PUAs derivatized with PFBHA (25 mM, Alfa Aesar) and extracted using hexane. Benzaldehyde (1 mM, Spex Certiprep LLC) was used as a surrogate standard and hexadecane-*d*34 (0.226 mg/mL, Sigma-Aldrich) as a recovery standard. Samples were analyzed via gas chromatography with mass spectral detection (GC-MS) analysis.

The GC instrument used was a HP 6890 gas chromatograph with an Agilent 7683 autosampler coupled to an HP 5973 quadrupole mass spectrometer. Samples were injected in splitless mode and separated on an Agilent HP-5MS column (30 m, 0.25 mm internal diameter, 0.25 µm film thickness) programmed at 60°C (2-minute hold), ramped to 240°C at 8°C/min, then to 285°C at 15°C/min. Helium was the carrier gas at a constant flow of 1.5 mL/min. Using selective ion monitoring mode, PUA identification was performed using their molecular ions: *m/z* 305 (heptadienal), 319 (octadienal), and 347 (decadienal). Other monitored ions included: *m/z* 57 (alkanes), 66 (*n-*hexadecane-*d*34), 181 (all PFBHA-derivatized aldehydes), 276 (all PUA molecules), and 301 (benzaldehyde). PUAs were quantified using response factors and comparing the benzaldehyde internal standard peak area to each PUA molecular ion. Method detection and quantitation limits for individual PUA molecules ranged from ~5.5 to 8.6 pmol and 17.5 to 28.6 pmol, respectively [28].

### Statistics

Two-factor ANOVAs were used to assess differences in PUA production between *p*CO_2_ treatments and the specific PUA molecules. Tests included 1) production of individual PUA molecules across *p*CO_2_ concentration in *S. marinoi*, 2) production of individual PUA molecules across *p*CO_2_ concentration in *F. pseudonana*, and 3) production of total PUA molecules across *p*CO_2_ concentration in both species. Prior to each analysis, the assumptions of normality and equal variance were tested using a Shapiro-Wilk test and Levene’s test using α = 0.05. Following these tests, the data was deemed non-normal (p < 0.05) and had unequal variance (p < 0.05). To satisfy the equal variance assumption, log transformations were performed for tests 1 and 2, while a square root transformation was performed for test 3 due to combining both groups of total PUA data. Data for tests 2 and 3 were still non-normal (p < 0.05) after transformations. However, since ANOVA is robust to non-normality, statistical analysis was performed using the transformed data. If significance was observed, Tukey’s *post-hoc* tests were performed to assess differences across factors and their respective effect sizes calculated. Statistical analyses were performed in R.

## Results and discussion

### Carbonate chemistry

**Fig 1:**
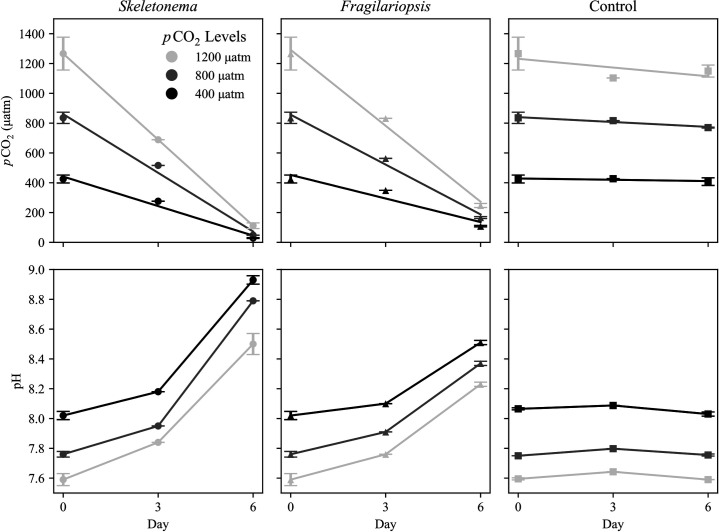
*p*CO_2_ drawdown by diatoms and pH of growth media over time. Colors show different *p*CO_2_ concentrations (µatm) with different symbols indicating the diatom species (*Skeletonmena*, *Fragilariopsis*, control). Trendline slope was used to infer daily *p*CO_2_ uptake rate. *p*CO_2_ concentrations and pH measurements on Day 0 (equilibrated media) and Day 6 are means of quadruplicate and triplicate measurements. Error bars represent standard deviations. Day 3 values are from single measurements.

*p*CO_2_ concentrations in the equilibrated media were slightly higher (4–6%) than target values of 400, 800, and 1200 μatm (Fig 1; Table 1). *p*CO_2_ concentration in diatom cultures decreased and pH increased over time. In *S. marinoi* cultures, *p*CO_2_ concentrations decreased >90% from starting *p*CO_2_ (Fig 1) and pH increased by approximately 1 pH unit (Fig 1). In *F. pseudonana* cultures, *p*CO_2_ decreased by 75–81% (Fig 1) and pH increased by about 0.5 pH units in each treatment (Fig 1). No change in pH or *p*CO_2_ was observed in the control bottles (Fig 1), indicating changes in DIC and pH were the result of diatom biology rather than outgassing from the experimental bottles.

**Table 1:** Water chemistry of growth media and diatom cultures over time.

[*p*CO_2_] (μatm)	Salinity (ppt)	pH	DIC (μmol/kg)	*p*CO_2_ (μatm)
**Equilibrated Media**				
400	32.13	8.02 ± 0.03	2034 ± 1	430 ± 30
800	32.15	7.76 ± 0.02	2141 ± 2	840 ± 40
1200	32.21	7.59 ± 0.04	2191 ± 3	1300 ± 100
***S. marinoi*** Day 3				
400	32.38	8.18	1980	276
800	32.40	7.95	2094	516
1200	32.46	7.84	2143	689
Day 6				
400	32.30 ± 0.04	8.93 ± 0.03	1619 ± 0	29 ± 3
800	32.33 ± 0.01	8.79 ± 0.00	1777 ± 6	48 ± 0
1200	32.36 ± 0.04	8.50 ± 0.06	1802 ± 2	110 ± 20
***F. pseudonana*** Day 3				
400	32.37	8.10	2009	349
800	32.33	7.91	2068	563
1200	32.39	7.76	2116	832
Day 6				
400	32.4 ± 0.1	8.51 ± 0.02	1822 ± 4	108 ± 5
800	32.4 ± 0.0	8.37 ± 0.01	1910 ± 9	166 ± 6
1200	32.5 ± 0.1	8.23 ± 0.02	1990 ± 9	250 ± 10

Day 0 growth media and Day 6 values are means ± standard deviations of quadruplicate and triplicate measurements. Day 3 values are single measurements.

The total amount of *p*CO_2_ drawn down over the six days by the two diatom species was slightly higher in *S. marinoi*. However, when normalized to chlorophyll biomass, *F. pseudonana* consumed more *p*CO_2_ (Fig 1; Table 1). *S. marinoi* consumed about 66, 131, and 193 μatm *p*CO_2_ per day in the 400, 800, and 1200 μatm treatments, while *F. pseudonana* consumed about 53, 112, and 170 μatm *p*CO_2_ per day. Both species consumed more carbon as *p*CO_2_ concentration increased. The observed increase in carbon uptake under elevated *p*CO_2_ did not elevate PUA production (see below), nor did the excess DIC uptake translate to differences in chlorophyll biomass or growth rates between *p*CO_2_ treatments (Table 2). This suggests that rather than stimulating population growth through high binding efficiency between RubisCO and *p*CO_2_ [36], the excess carbon being fixed in the high *p*CO_2_ treatments was being allocated to carbon pools other than particulate biomass production. This finding has been observed in other studies exploring the relationship between elevated *p*CO_2_ and phytoplankton production, where excess *p*CO_2_ caused an increase in the release of dissolved carbohydrates that, in turn, precipitated into transparent exopolymers [46–50]. In our experiments, inorganic phosphorus and nitrogen were added in excess. Thus, the increased DIC that was taken up by the diatoms in the medium and high *p*CO_2_ treatments likely generated exopolymer carbohydrate production with high C:P and C:N ratios [51]. If true, the increased precipitation of these carbohydrates into aggregations augments the already active role of diatom production during upwelling events.

**Table 2:** Total chlorophyll-a (μg) and average specific growth rates (d^-1^) over the 6-day experiment.

Sample	Total *S. marinoi* chlorophyll-*a* (μg)	*S. marinoi* Specific Growth Rate	Total *F. pseudonana* chlorophyll-*a* (μg)	*F. pseudonana* Specific Growth Rate
Inoculum	0.87 ± 0.2	-----	1.99 ± 0.09	-----
400 µatm	98.02 ± 6.3	0.79 ± 0.01	66.43 ± 6.6	0.58 ± 0.02
800 µatm	105.42 ± 21.5	0.80 ± 0.03	72.78 ± 17.1	0.60 ± 0.04
1200 µatm	96.19 ± 11.2	0.78 ± 0.02	80.03 ± 38.1	0.60 ± 0.08

Errors are standard deviations based on quadruplicate replication. No significant differences were found within species for growth rates and total chlorophyll-*a* across *p*CO_2_ concentrations.

### PUA production

**Fig 2:**
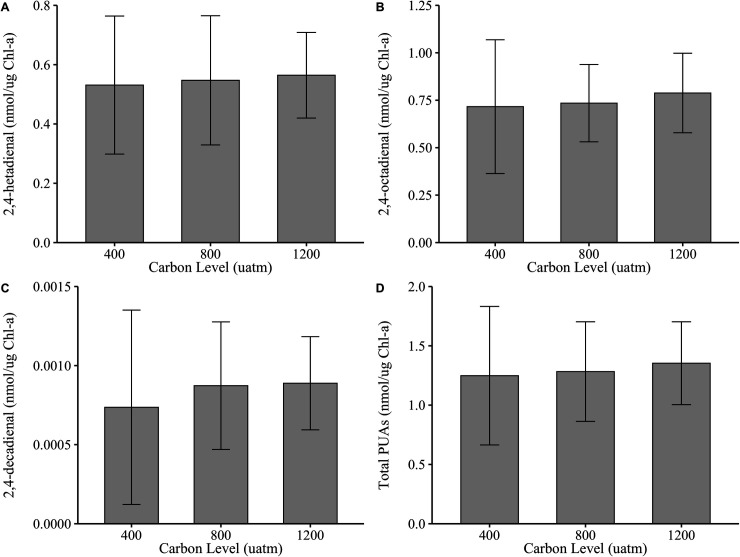
*S. marinoi* PUA mass (nmol/μg chlorophyll-*a*) across *p*CO_2_ concentration (µatm) for (A) 2,4-heptadienal, (B) 2,4-octadienal, (C) 2,4-decadienal, and (D) total PUAs. Gray bars are means of triplicate treatment cultures and error bars show 95% confidence intervals. No significant difference in total PUA molecule production was observed across *p*CO_2_ concentration (p = 0.294). Note different Y axis scales.

**Fig 3:**
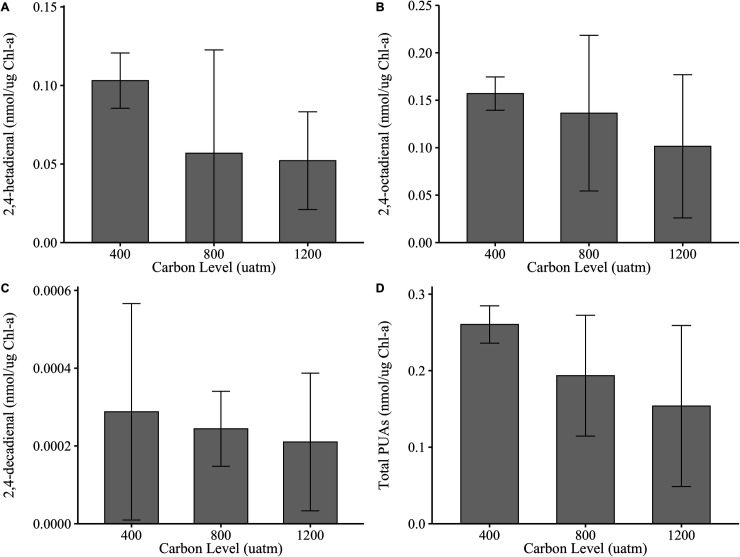
*F. pseudonana* PUA mass (nmol/μg chlorophyll-*a*) across *p*CO_2_ concentration (µatm) for (A) 2,4-heptadienal, (B) 2,4-octadienal, (C) 2,4-decadienal, and (D) total PUA production. Gray bars are means of replicate treatment cultures and error bars show 95% confidence intervals*. F. pseudonana* produced significantly more PUAs in the 400 μatm treatment than the 1200 μatm treatment (p = 0.028). Values below the quantification limit were quantified and visualized using MQL/2. Note Note different Y axis scales.

There was no evidence that *p*CO_2_ concentration affected the total production of PUA molecules for *S. marinoi* (*p *= 0.294), with concentrations ranging from 1.25 nmol/μg chlorophyll-*a* to 1.35 nmol/μg chlorophyll-*a* across treatments (Fig 2D). There was, however, strong evidence that individual PUAs were produced at significantly different concentrations (e.g., heptadienal was observed at higher concentrations than decadienal), but this was independent of *p*CO_2_ concentration (p << 0.001; Fig 2A–C). There was no evidence suggesting an interaction between individual PUA molecule production and *p*CO_2_ concentration (p = 0.903). PUA production in *F. pseudonana* was affected by *p*CO_2_ concentration (p = 0.026, η^2^ = 0.006; Fig 3A), whereby higher production of PUAs was observed in the 400 μatm *p*CO_2_ treatment compared to 1200 μatm treatment (Tukey’s *post-hoc,* p = 0.028). While the effect size of *p*CO_2_ concentration on PUA production in *F. pseudonana* was small, lower PUA production under elevated *p*CO_2_ has been observed previously [40]. Studies suggest that a possible reason for this is a reduction in precursor PUFA molecules under high *p*CO_2_ [39]. In particular, the PUFA eicosapentaenoic acid, a precursor PUA molecule [52], was produced at slightly lower concentrations under elevated *p*CO_2_ [53]. However, replication of this experiment with increased sample sizes may increase confidence in the trends seen here.

Like *S. marinoi*, individual PUAs were produced at significantly different concentrations by *F. pseudonana* (e.g., octadienal was observed at higher concentrations than decadienal), independent of *p*CO_2_ concentration (p << 0.001; Fig 3A–C). There was no evidence suggesting an interaction between individual PUA molecule production and *p*CO_2_ concentration (p = 0.594). However, *S. marinoi* produced significantly higher concentrations of individual PUAs than *F. pseudonana*, ranging from five- to nine-times higher depending on *p*CO_2_ concentration (p << 0.001; Figs 2 and 3). Diatom species had a very large effect on the results, explaining 94.9% (η^2^ = 0.949) of the observed variance. This is not surprising given that PUFA precursor molecules are known to vary by species and habitat. In a survey of 10 diatoms and seven dinoflagellates, phytoplankton group explained 46% of the variance in fatty acid profiles, whereas habitat explained 31% of the variance [54]. Direct comparison of PUA concentrations produced by *S. marinoi* in the current study to data from previous studies is confounded by our use of chlorophyll standardization, as cell counts are traditionally used. However, total PUA production by *F. pseudonana* under ambient *p*CO_2_ closely aligns with previous work using the same species [28].

PUAs are widely considered to be grazing deterrents [19–20], and elevated diatom productivity and community dominance following upwelling events may result from the allelopathic properties of PUAs. However, our results suggest that PUAs are not produced in greater concentrations under elevated *p*CO_2_ despite their carbon-based composition. Further, seasonal upwelling cycles are likely not impacting trophic dynamics by way of PUA production and a resultant decrease in grazer fecundity.

Not explored in this study is the potential synergy between *p*CO_2_ concentration and nutrient limitation with respect to PUA production. Seasonal upwelling delivers elevated *p*CO_2_ concentrations and biologically active inorganic nutrients to surface waters. As climate change intensifies, however, oceanic *p*CO_2_ concentrations are expected to increase independent of the seasonal delivery of these nutrients through upwelling [55]. PUA production is known to be impacted by nitrogen and phosphorus availability, likely because of their importance to protein and enzyme formation [29,30,56]. For example, lipoxygenase enzymes are needed to process PUA-precursors and form PUA molecules; limiting protein-building nutrients would impact enzyme expression and, in turn, PUA production [18]. Future studies should assess PUA production under climate change scenarios, including elevated *p*CO_2_ and limiting nutrients in the mixed layer [57]. In addition, other diatom species could be tested beyond marine, chaining species, including more benthic diatoms. These studies would help us understand PUA production and diatom trophic dynamics under *p*CO_2_ concentrations in current and future oceans.

## Supporting information

S1 File S1. pH data of treatments over the 6-day experiment of blank, *S. marinoi*, and *F. pseudonana* bottles. ‘Level’ indicates target *p*CO_2_ concentration (µatm), ‘pH’ indicates the averaged pH measurement, and ‘sd’ indicates the standard deviations of the pH measurements. Day 0 growth media and Day 6 values are means ± standard deviations of quadruplicate and triplicate measurements. Day 3 values are single measurements. S2. DIC data of treatments over the 6-day experiment of blank, *S. marinoi*, and *F. pseudonana* bottles. ‘Level’ indicates target *p*CO_2_ concentration (µatm), ‘Carbon Level’ indicates the averaged *p*CO_2_ measurement, and ‘sd’ indicates the standard deviations of the *p*CO_2_ measurements. Day 0 growth media and Day 6 values are means ± standard deviations of quadruplicate and triplicate measurements. Day 3 values are single measurements. S3. PUA data of treatments over the 6-day experiment of *S. marinoi*, and *F. pseudonana* bottles. ‘Level’ indicates target *p*CO_2_ concentration (µatm), while ‘Hepta,’ ‘Octa,’ ‘Deca,’ and ‘Total’ indicate the averaged PUA measurements (nmol/μg chlorophyll-*a*) of heptadienal, octadienal, decadienal, and total PUAs.
(ZIP)
